# Durability in recreational runners: effects of 90-min low-intensity exercise on the running speed at the lactate threshold

**DOI:** 10.1007/s00421-024-05631-y

**Published:** 2024-10-09

**Authors:** Olli-Pekka Nuuttila, Vesa Laatikainen-Raussi, Krista Vohlakari, Iida Laatikainen-Raussi, Johanna K. Ihalainen

**Affiliations:** 1 Faculty of Sport and Health Sciences, University of Jyväskylä, Jyväskylä, Finland; 2 The UKK Institute for Health Promotion Research, Kaupinpuistonkatu 1, 33500 Tampere, Finland; 3 Finnish High Performance Institute KIHU, Rautpohjankatu 6, Jyväskylä, Finland

**Keywords:** Physiological resilience, Fatigue, Fatigue resistance, Endurance performance

## Abstract

**Purpose:**

Recent studies have suggested that the capability to resist deterioration of physiological characteristics could be an independent factor contributing to endurance performance. This study aimed at investigating whether prolonged low-intensity exercise induces shifts in the lactate threshold, and whether fatigue-induced changes differ between the sexes.

**Methods:**

A total of 31 (15 females) recreational runners performed an incremental treadmill test and a 90-min low-intensity exercise (LIT90) on two separate occasions. The LIT90 was performed at 90% of the first lactate threshold speed (LT1v), derived from the incremental treadmill test. The LT1v was determined from a 5-stage (3 min) submaximal threshold test (SubmaxLT), performed before and after LIT90. The SubmaxLTs were followed by a 10/5 reactivity jump test. Respiratory gases, heart rate (HR), and HR-derived detrended fluctuation analysis alpha 1 (DFA-a1) were assessed every 15 min during the LIT90.

**Results:**

A significant decrease (*p* < 0.01) was observed in the LT1v in females (− 5.8 ± 4.4%) and in males (− 5.3 ± 6.4%). The HR increased (*p* < 0.001) similarly in females (5.9 ± 3.1%) and in males (5.5 ± 3.6%) during the LIT90, while energy expenditure increased (3.1 ± 4.5%, *p* = 0.013) in females but remained unchanged in males (0.9 ± 3.1%). Change in DFA-a1 during the LIT90 was the only marker that correlated significantly with the relative change of LT1v (*r* = 0.463, *p* = 0.013).

**Conclusion:**

LIT90 induced significant decreases in the LT1v, and the changes were comparable between sexes. DFA-a1 could be a potential intra-session marker of durability.

## Introduction

The main determinants of endurance performance have traditionally been summarized into three major factors: maximum oxygen uptake, fractional utilization of VO_2_ (lactate/ventilatory threshold), and exercise economy (Bassett and Howley 2000). The golden standard method for the assessment of these capabilities is the maximal incremental test, which is typically performed at well-rested and fully recovered state. However, it has been suggested that assessing the changes in these parameters after prolonged exercise could provide more comprehensive information on the long-distance performance (Noordhof et al. 2021).

During recent years, the concepts of “durability” (Maunder et al. 2021) or “physiological resilience” (Jones 2023) have been proposed as potential factors contributing significantly to long-distance endurance performance. Durability has basically been defined as an individual’s capability to resist and delay deteriorations (magnitude and time of onset) in physiological profiling characteristics during prolonged exercise (Maunder et al. 2021). In practice, the capability has been assessed through changes in physiological parameters, such as heart rate (HR) (Matomäki et al. 2023; Smyth et al. 2022), HR variability (HRV) (Gronwald et al. 2021; Matomäki et al. 2023), or energy expenditure (EE) (Matomäki et al. 2023) during long-duration exercise. Other assessment options have included changes in threshold (Gallo et al. 2024; Hamilton et al. 2024; Stevenson et al. 2022) or maximal performance (Hamilton et al. 2024; Noordhof et al. 2021) after prolonged endurance exercise. Recently, Stevenson et al. (2022) found that 2 h of low-intensity cycling did not increase energy expenditure (EE) significantly, whereas the power at the ventilatory threshold decreased by about 10%. It is also noteworthy that significant associations have been found with fatigue-induced changes in threshold performance and time-to-task failure (Gallo et al. 2024) as well as change in the maximum 5-min time-trial performance (Hamilton et al. 2024), highlighting the practical relevance of threshold decrements.

As the concept of durability is still quite new, there are no studies that have examined the changes in metabolic thresholds after prolonged running exercise. Since the biomechanics and muscular load of running differ significantly from cycling (Sandbakk et al. 2021), it could be hypothesized that the fatigue patterns may also differ. For example, Brownstein et al. (2022) have suggested that running-based endurance exercise would induce greater impairments in the nervous system function, while cycling-based endurance exercise induces impairments in the contractile functions of the muscles, which could potentially have implications also on the durability. Another aspect that has not yet been examined is the possible difference between the sexes. Some studies have suggested that females may have greater “durability”, namely lower magnitude and later occurring decoupling of HR during marathon (Smyth et al. 2022) and less neuromuscular fatigue after strenuous exercise protocols (Ansdell et al. 2019, 2020) or trail-running races (Besson et al. 2021; Temesi et al. 2015). In support of potential sex differences, the analyses of Le Mat et al. (2023) suggested that the longer the running distance in trail-running competitions, the smaller the gap between males and females. The purpose of this study was to examine the effects of prolonged low-intensity running exercise on physiological responses and lactate threshold performance. Furthermore, the aspiration was to find out whether the fatigue-induced changes differ between sexes. Finally, the study analyzed associations between exercise-induced shifts in physiological variables and changes in lactate threshold performance.

## Methods

### Participants

The data collection was performed as part of an ongoing study “Performance variation and monitoring in recreationally active runners” that monitors training, health, and recovery of recreational runners over a year. A total of 36 (17 females, 18 males) recreational runners were recruited to participate in the study. The participants were training regularly (> 250 h/year), and were healthy and free of any chronic diseases. In the current dataset, five participants were excluded from the analyses due to: 1) prolonged gap (> 3 weeks) between the incremental treadmill test and the durability test (*n* = 4) and 2) not performing the durability test (*n* = 1). The baseline characteristics of the participants included in the analyses are presented in Table 1. An informed consent was obtained from all individuals participating in the study. The study protocol was approved by the ethics committee of the University of Jyväskylä (534/13.00.04.00/2023).

**Table 1:** Baseline characteristics of the participants

	Females (*n* = 15)	Males (*n* = 16)
Age	36 ± 9	36 ± 7
Height	168 ± 7	180 ± 5
Body mass	62 ± 7	78 ± 10
VO_2max_	50.1 ± 7.3	52.7 ± 8.0

### Study protocol

The study protocol consisted of two separate test sessions: 1. An incremental treadmill test during which the first lactate threshold (LT1) was analyzed 2. A durability test consisting of a 90-min low-intensity exercise (LIT90) which was followed and preceded by a submaximal 5-stage (3 min) threshold assessment test (SubmaxLT). The testing sessions were planned to be performed on two consecutive days. However, due to scheduling issues, only 18 participants were able to perform the test on consecutive days, and therefore, the average gap was 3.4 days. Since the main outcome parameter results (LT, HR, energy expenditure) were not affected by the gap (1 day vs. > 1 day), all participants that performed the tests within 3 weeks were included in the analyses. Three weeks was chosen as a cut-off, because a training period of this length has not previously induced adaptations in the lactate threshold in recreational runners (Zinner et al. 2018).

### Incremental treadmill test

An incremental treadmill test until volitional exhaustion was performed on a motorized treadmill (H/P/Cosmos Saturn 300/100 r). The test consisted of 3-min stages with constant speed and incline (1.0%). The treadmill was stopped after each stage for blood lactate sampling from the fingertip (Biosen S_line Lab + lactate analyzer, EKF Diagnostic, Magdeburg, Germany), after which the speed was increased by 1 km/h from the previous stage. Respiratory gases (Jaeger VyntusTM CPX, CareFusion Germany 234 GmbH, Hoechberg, Germany) and HR (Polar H10, Polar Electro Oy, Kempele, Finland) were monitored throughout the test. The maximum oxygen uptake (VO_2max_) was defined as the highest 60 s average of VO_2_.

The LT1 was set to the point where blood lactate increased for the first time by 0.3 mmol/l from the lowest value during the test (Nuuttila et al. 2020; Vesterinen et al. 2016). The reliability of the method has not been published previously, but in the current data set, the coefficient of variation between the incremental treadmill test-derived LT1 and the pre-SubmaxLT-derived LT1 was 4.0%, and the intraclass correlation coefficient (1,3) was 0.93, suggesting comparable reliability of previously reported threshold assessment methods in well-trained athletes (Pallarés et al. 2016).

### Durability test

**Fig. 1:**
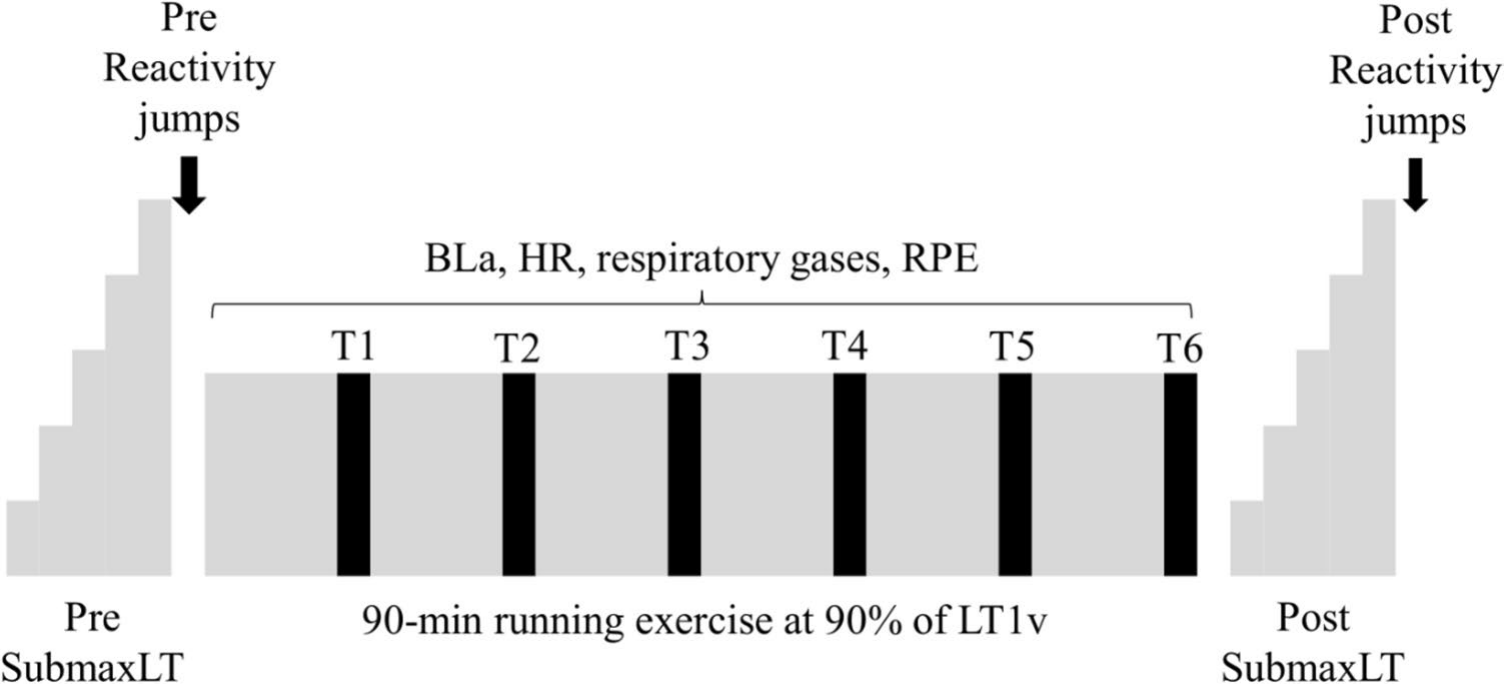
Protocol of the durability test

The durability test (Fig. 1) was adapted from the protocol used by Stevenson et al. (2022). The test session started with a pre-SubmaxLT, followed by a 10/5 reactivity jump test (Southey et al. 2023). After that, a 90-min low-intensity running exercise (LIT90) was performed, and just like before the exercise, it was followed by a post-SubmaxLT and reactivity jump tests. The SubmaxLT and LIT90 were performed on the same treadmill as the incremental treadmill test. Body mass was measured before the pre-SubmaxLT and after the post-SubmaxLT. The participants were allowed to drink water ad libitum during LIT90.

The LIT90 was run at a speed that was 90% of the individual’s LT1v in the incremental treadmill test. The speed and incline (1.0%) were constant throughout the exercise. The treadmill was stopped for blood lactate sampling and attaching of the mask every 15th minute (0:10, 0:25, 0:40, 0:55, 1:10, 1:25). After that, respiratory gases (VO_2_, ventilation, and respiratory exchange ratio) were recorded for five consecutive minutes (T1-T6, Fig. 1), and the average of the two last minutes was used in the analyses. From these results, also EE (kcal/min) was derived with a formula: VO_2_ (l/min)*(1.2064*RER + 3.8455) and the result was converted to kJ/min by multiplying the number by 4.186 (Keskinen et al. 2004). HR was monitored throughout the exercise with a sensor (Polar H10), and the results were analyzed for the same 2-min windows as for the respiratory parameters. In addition to HR, detrended fluctuation analysis alpha 1 (DFA-a1) was analyzed with Kubios HRV Scientific software (version 4.0). Default settings were used in accordance with Van Hooren et al. (2023), and automatic beat correction (Lipponen & Tarvainen 2019) was applied for the data. Three individuals had higher artifact percentage than the suggested within-participant limit of 3% (Rogers et al. 2021); thus, two females and one male were excluded from the DFA-a1-related analyses.

The SubmaxLT consisted of 3-min stages that were defined based on the incremental treadmill test results. The starting speed of the test was set to 2 km/h below the LT1v. Similar to the incremental treadmill test, the speed increased by 1 km/h after each stage and blood lactate samples were collected from the fingertip, while the incline was kept constant (1.0%). HR and respiratory gases were monitored throughout the test, and the last 60 s (respiratory variables) and 30 s (HR) averages of each stage were used in the analyses. In addition, the rating of perceived exertion (RPE) was assessed with a 6–20 scale (Borg 1982) at the end of each stage. The LT1 (speed, VO_2_, EE, ventilation, HR, and RPE) was analyzed from the test with the same criteria as in the incremental treadmill test. In case the threshold occurred between the stages, all results were interpolated to the exact speed of the LT1.

Reactivity jump (RJ power) tests were performed one minute after the SubmaxLT on a custom-built contact mat. In the test, the participants performed ten repeated jumps explosively with minimal contact time. Their hands were held on the hips, and minimal bending on the knees was allowed. Contact times and flight times were derived from the contact mat, and the power of each jump was calculated using the formula of Bosco et al. (1983). The average of the five best jumps was used in the analyses.

### Statistical analyses

The results are presented as mean ± standard deviation (SD). The normality of the variables was assessed with the Shapiro–Wilk test. The differences between time points and the interaction between time × sex were examined either with repeated-measures ANOVA (multiple time points), with LSD post hoc test, or with paired or independent samples t test (two time points). The Pearson correlation coefficient was used to analyze associations between T1 and T6 changes of monitored physiological variables and pre-post relative change of LT1v. Statistical analyses were performed with IBM SPSS Statistics v.28 program (SPSS Inc, Chicago, IL, USA).

## Results

### 90-min low-intensity exercise

Descriptive characteristics of the 90LIT are presented in Table 2. The characteristics did not differ between sexes except for the speed relative to maximum (greater in females, *p* = 0.017) and the absolute blood lactate values after the exercise (greater in males, *p* = 0.045). Weight loss during the exercise averaged − 1.6 ± 0.5% in females and − 2.2 ± 1.0% in males with a significant between groups difference (*p* = 0.042).

**Table 2:** Characteristics of the 90-min low-intensity exercise (LIT90). Blood lactate and RPE values are from the end of the exercise (T6)

	Females (*n* = 15)	Males (*n* = 16)
Speed (km/h)	9.6 ± 1.5	10.0 ± 1.5
Speed (%/vmax)	63.9 ± 3.2	60.5 ± 4.1
VO_2_ (ml/kg/km)	216 ± 12	218 ± 20
HRavg (%/max)	79.5 ± 4.7	78.0 ± 5.4
HRpeak (%/max)	85.4 ± 4.0	84.3 ± 5.1
Blood lactate (mmol/l)	1.17 ± 0.40	1.48 ± 0.41
RPE (6–20)	12.8 ± 2.1	13.2 ± 1.3

*VO*_*2*_ oxygen consumption as an average of T1–T6, *HR* heart rate, *RPE* rating of perceived exertion

**Fig. 2:**
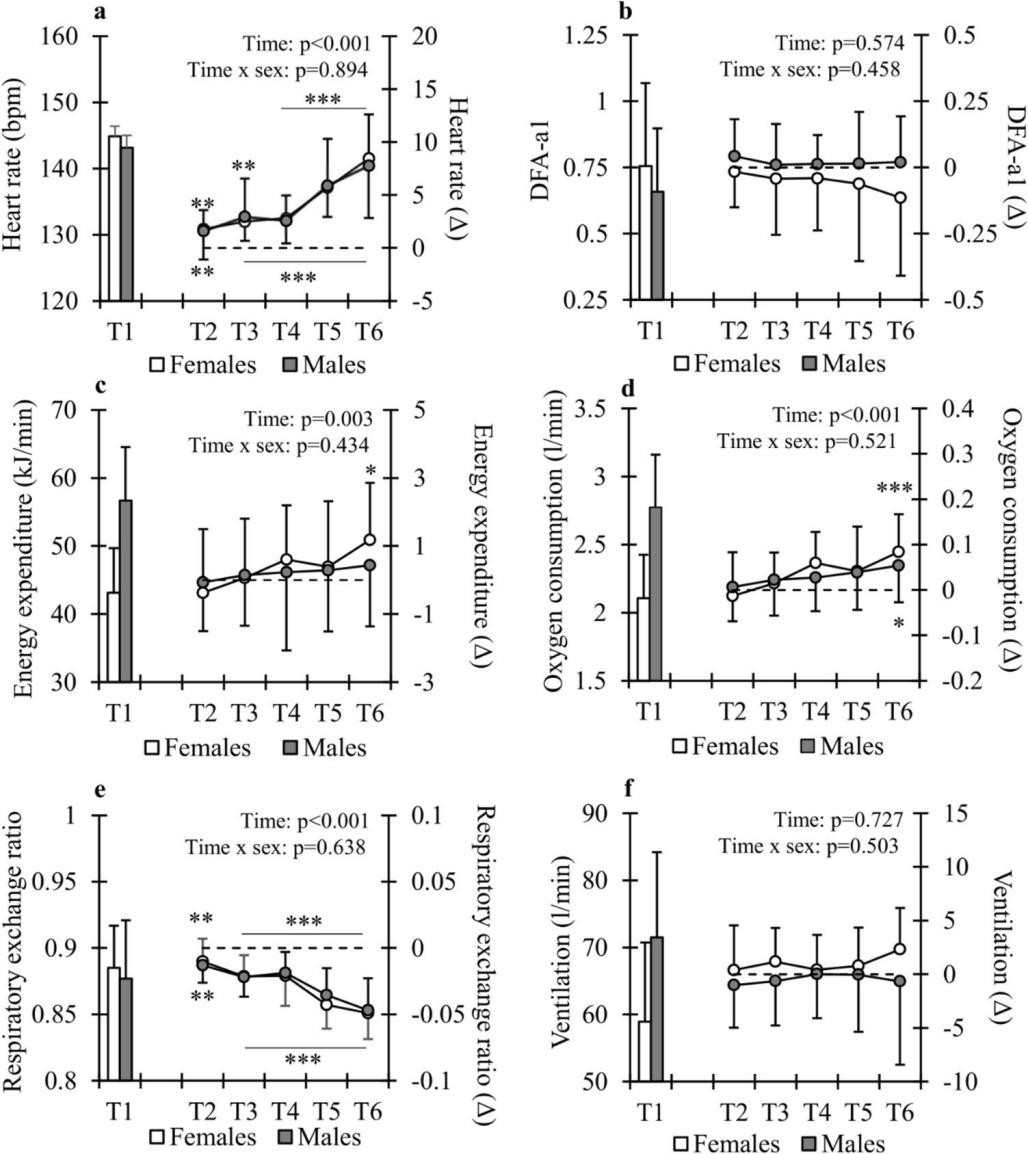
Changes in physiological variables monitored during the LIT90. T1–T6 refer to the 15-, 30-, 45-, 60-, 75-, and 90-min timepoints. *p < 0.05, **p < 0.01, ***p < 0.001 compared with T1. Lines in figures a and e indicate that all respective changes were significant compared with T1

Changes in physiological variables monitored during the exercise are shown in Fig. 2. Significant changes were found in HR which increased from T1 to T6 on average by 5.9 ± 3.1% in females and by 5.5 ± 3.6% in males. In addition, RER decreased (*p* < 0.001) and oxygen consumption increased (*p* < 0.013) in both sexes from T1 to T6. EE increased from T1 to T6 only in females (3.1 ± 4.5%, *p* < 0.05), while it remained unchanged in males (0.9 ± 3.1%, *p* = 0.319).

### Changes in the first lactate threshold and reactivity jump performance

**Fig. 3:**
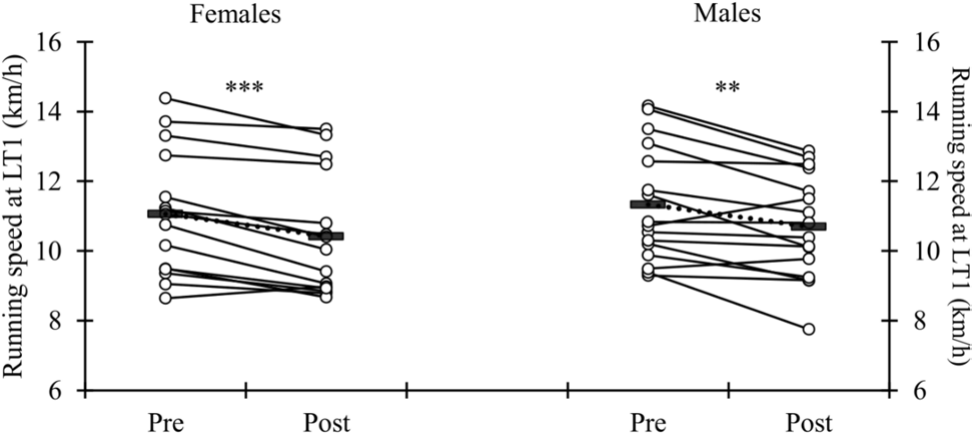
Speed of the first lactate threshold (LT1v) before (Pre) and after (Post) LIT90. **p < 0.01, ***p < 0.001 Pre–Post-difference

There was a significant decrease in the LT1v after the 90LIT in females (− 5.8 ± 4.4%, *p* < 0.001) and in males (− 5.3 ± 6.4%, *p* = 0.003), and the magnitude of decrease was similar in both sexes (Fig. 3). EE and blood lactate concentrations at the LT1 decreased (*p* < 0.05), and HR increased (*p* < 0.001) in both sexes, but RPE increased (*p* < 0.001) only in males (Table 3). Ventilation at the LT1 was the only marker that was not different between pre and post in either of the sexes. The average power (females: 24.3 ± 6.5 vs. 23.4 ± 7.5 W/kg; males 28.1 ± 8.3 vs. 26.8 ± 8.8 W/kg), contact time (females: 170 ± 18 vs. 175 ± 27 ms; males: 193 ± 49 vs. 208 ± 73 ms), and jump height (females: 21.2 ± 4.6 vs. 20.7 ± 5.2 cm; males: 27.0 ± 6.1 vs. 26.9 ± 6.3 cm) of the reactivity jump test remained unchanged in both sexes after the 90LIT.

**Table 3:** Pre–Post-comparison of measured variables at LT1

	Females (*n* = 15)		Males (*n* = 16)	
	LT1 Pre	LT1 Post	LT1 Pre	LT1 Post
Speed (km/h)	11.1 ± 1.8	10.4 ± 1.8***	11.3 ± 1.7	10.7 ± 1.5**
HR (bpm)	152.4 ± 13.1	158.5 ± 11.6***	145.6 ± 8.8	154.9 ± 12.1***
VO_2_ (l/min)	2.42 ± 0.39	2.35 ± 0.35*	3.11 ± 0.52	3.01 ± 0.40
EE (kJ/min)	49.9 ± 8.0	47.7 ± 7.1***	63.8 ± 10.8	60.8 ± 8.2*
VE (l/min)	67.2 ± 13.5	67.1 ± 12.4	76.5 ± 16.8	76.2 ± 14.4
Bla (mmol/l)	1.35 ± 0.32	1.20 ± 0.24*	1.65 ± 0.45	1.32 ± 0.35*
RPE (6–20)	12.4 ± 1.4	13.2 ± 1.7	12.3 ± 1.7	14.0 ± 1.2***

*p < 0.05, **p < 0.01, ***p < 0.001 compared with pre

### Exercise-induced changes in physiological markers and changes in threshold performance

**Fig. 4:**
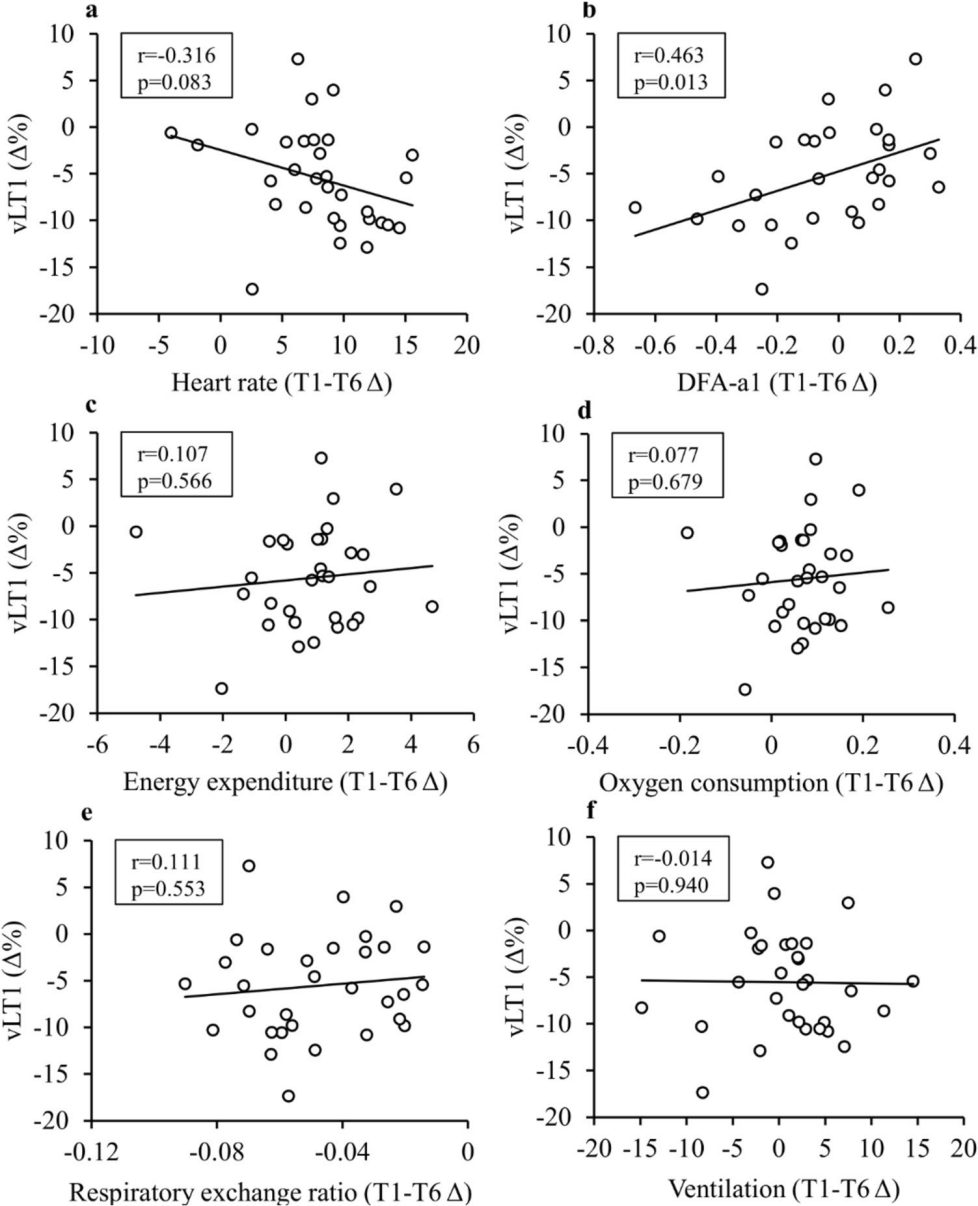
Pearson correlation coefficients between the change of the first lactate threshold speed (LT1v) and intra-session T1–T6 changes of physiological variables

When the associations between intra-session T1–T6 changes of physiological variables and the relative change of LT1v were analyzed in the whole study group (Fig. 4), the only variables that exceeded a correlation coefficient of 0.2 were the change in HR (− 0.316, *p* = 0.083) and DFA-a1 (0.463, *p* = 0.013).

## Discussion

The key finding of this study relates to the significant decrease in LT1v that was observed after the LIT90. The magnitude of change in this case was similar in both sexes. Although HR and oxygen consumption increased during the exercise, these changes were not associated with the change in threshold performance. Non-linear heart rate variability, DFA-a1, did not change during the exercise, but the change correlated with the relative change of LT1v, suggesting its potential usefulness in monitoring of fatigue during the training sessions.

Although exercise-induced fatigue is a well-known phenomenon (Abbiss & Laursen 2005), the concepts of durability (Maunder et al. 2021) and physiological resilience (Jones 2023) have been proposed only recently to describe the capability in more detail. Changes in ventilatory thresholds (Gallo et al. 2024; Hamilton et al. 2024; Stevenson et al. 2022) or critical power (Clark et al. 2019a, b; Spragg et al. 2023, 2024) have been examined among cyclists, but to our best knowledge, no similar studies have been performed previously in runners. Therefore, the current study provided novel findings regarding the durability in recreational runners.

In line with the observations in cyclists (Gallo et al. 2024; Hamilton et al. 2024; Stevenson et al. 2022), the current study found a significant decrease in lactate threshold performance after prolonged low-intensity exercise. Interestingly, the magnitude of change was almost identical with the 6% decrease in ventilatory threshold reported by Hamilton et al. (2024) after 150-min low-intensity cycling, and the approximate 5% mean decrease was the same that was used as a cut-off value for significant ventilatory threshold decrease in the study by Gallo et al. (2024). As the mechanical loading of running differs from cycling (Sandbakk et al. 2021), it was anticipated that the changes in LT1 could be even greater than those of cycling. While current threshold impairments occurred after exercise that was 1 h shorter than has been reported in cycling (Hamilton et al. 2024), there could indeed be differences in the decrements of threshold performance. In comparison to Stevenson et al., (2022), who found 10% decrease after 2-h cycling, it is important to acknowledge that in their study, participants performed the exercise after 10-h overnight fasting, which likely promoted the fatigue-induced changes. To conclude, the phenomenon that threshold performance decreases seem not to be discipline-specific but concerns comparably cyclists and runners.

During LIT90, there was no significant increase in blood lactate, and it seems that the participants remained within low-intensity zone. However, as indicators of accumulating fatigue, increase in HR and VO_2_ was found in both sexes. As expected, also RER decreased, implying higher reliance on fat oxidation. When physiological characteristics were examined at the lactate threshold before and after LIT90, ventilation was the only parameter that did not change significantly, in line with Stevenson et al. (2024). This demonstrates the challenges in HR- or RPE-based intensity prescriptions, as the threshold anchors can change during exercise. An interesting observation was that there occurred no negative changes in EE or VO_2_ until the final time point (T6) of LIT90. This highlights how the duration of exercise needs to be sufficient before significant fatigue-induced changes can be assessed. Based on the current and previous studies (Gallo et al. 2024; Hamilton et al. 2024; Stevenson et al. 2022), 90–150-min exercises appear sufficient to induce changes in physiological characteristics. In this setup, longer duration or higher intensity exercise between SubmaxLT tests could have been too demanding, as there already were two participants who were not able to finish the final stage of the post-SubmaxLT.

Although it has been suggested that females could be more endurant than males in regard to HR decoupling during a marathon (Smyth et al. 2022) or neuromuscular fatigability after trail-running races (Besson et al. 2021; Temesi et al. 2015), the current study did not find significant differences in any of the primary outcomes. It has been speculated that potential explanatory mechanisms may relate to higher percentage of type 1 fibers and the ability to oxidize fat at given intensity (Besson et al. 2022; Tiller et al. 2021). It is possible that a relatively small sample and heterogeneous participant characteristics contributed to the lack of differences in the present study. Furthermore, it is possible that significant differences would require longer and more demanding exercise protocols. Le Mat et al. (2023) showed that for every additional 10 km in a trail-running competition, there is a 4% decrease in speed for males and a 3.3% decrease for females, suggesting that the performance gap between sexes may narrow with longer race distances. This could imply that the physiological differences between males and females become less pronounced over longer distances, potentially due to the endurance factors mentioned earlier.

For practical purposes, it would be very useful to find feasible indicators that could be used in live monitoring of fatigue/durability also in field conditions. As HR is monitored routinely by many endurance athletes, drift in HR could be a tempting parameter for that purpose. Maunder et al., (2021) and Smyth et al. (2022) have suggested that the decoupling of HR in relation to external load could be one potential marker for field-based estimations of durability. In the current study, the change in HR did not correlate significantly with the relative change of LT1v, partly due to one clear outlier (Fig. 4). Meanwhile, another HR-based variable, DFA-a1, was the only marker in which the change was associated with the impairment of LT1v. This aligns with the suggestions of Rogers and Gronwald (2022) and Van Hooren et al. (2023) who have proposed DFA-a1 as a potential indicator of exercise-induced fatigue. While HRV variables in the time and frequency domain primarily describe the activity of the parasympathetic nervous system and are already significantly reduced at low exercise intensities, the advantage of the DFA-a1 is its applicability even at high exercise intensities (Hautala et al. 2003). In practice, the DFA-a1 appears to decrease quite linearly with exercise intensity from moderate to severe intensity domains (Hautala et al. 2003; Rogers and Gronwald 2022). The changes in DFA-a1 observed in the present study are probably related to both a withdrawal of parasympathetic nervous system activity and an increase in sympathetic nervous system activity due to an increased internal demand of the exercise. In the future, it would be useful to further investigate autonomic nervous system function and changes in its regulation due to long-term exercise-induced fatigue. As a limitation, HRV is very sensitive to artifacts, and in the current study, three participants were excluded from the DFA-a1 analyses due to insufficient data quality. This challenge could limit the applicability of the parameter in the field and non-controlled use, or at least it calls for an efficient method to ensure sufficient data quality and dealing with the artifacts.

Although durability seems to be an important capability that is associated with long-distance endurance performance (Gallo et al. 2024; Hamilton et al. 2024), its determinants are still poorly understood. Jones (2023) suggested that the capability would be affected by the combination of loss of efficiency and decrease in VO_2max_. Furthermore, the fat oxidation capacity (Gallo et al. 2024) and carbohydrate availability (Clark et al. 2019a, b) have been suggested to have an impact on durability. Obviously, the testing protocol can have a significant effect on the outcome. There are yet important questions to be solved: for instance, whether durability should be assessed with competition-like carbohydrate-fueling or in a “challenged” condition (e.g., after overnight fasting). Most protocols have examined changes in threshold performance after low-intensity exercise (Gallo et al. 2024; Hamilton et al. 2024; Stevenson et al. 2022), while exercise intensity above the lactate threshold would be closer to competition intensity in most endurance disciplines. As the intensity of an exercise might be a more relevant factor than total work performed affecting negatively the performance (Spragg et al. 2024), and metabolic and neuromuscular determinants of fatigue development differ according to the intensity domain in which the exercise is performed (Black et al. 2017), this question should also be addressed in more detail in the future. Finally, the methods to assess fatigue (e.g., decrease in threshold performance vs. maximum performance vs. economy) should also be compared in more detail to understand their relevance regarding competition performance.

## Conclusions

In conclusion, the current results demonstrated significant impairment of LT1v after 90 min of low-intensity running exercise, and the magnitude of change was not different between sexes. Since the change in DFA-a1 correlated with the relative change in LT1v, it could be a potential marker for intra-session monitoring of fatigue. Further studies are needed to examine the most useful methods for assessing durability as well as potential contributors to the capability itself.

## Data Availability

The datasets generated and/or analyzed during the current study are available from the corresponding author on reasonable request.

## Abbreviations

DFA-a1: Detrended fluctuation analysis alpha 1
EE: Energy expenditure
HR: Heart rate
HRV: Heart rate variability
LIT90: 90-Min low-intensity exercise
LT1v: First lactate threshold speed
RPE: Rating of perceived exertion
VO_2_: Oxygen consumption
SubmaxLT: Submaximal threshold test
